# Modulatory effects of heparin and short-length oligosaccharides of heparin on the metastasis and growth of LMD MDA-MB 231 breast cancer cells *in vivo*

**DOI:** 10.1038/sj.bjc.6603928

**Published:** 2007-08-28

**Authors:** P Mellor, J R Harvey, K J Murphy, D Pye, G O'Boyle, T W J Lennard, J A Kirby, S Ali

**Affiliations:** 1 Breast Cancer Research Group, School of Surgical and Reproductive Sciences, Newcastle University, Newcastle-upon-Tyne, NE2 4HH, UK; 2 Department of Chemical and Biological Sciences, University of Huddersfield, Huddersfield, HD1 3DH, UK

**Keywords:** CXCL12, CXCR4, glycosaminoglycan, metastasis, breast cancer

## Abstract

Expression of the chemokine receptor CXCR4 allows breast cancer cells to migrate towards specific metastatic target sites which constitutively express CXCL12. In this study, we determined whether this interaction could be disrupted using short-chain length heparin oligosaccharides. Radioligand competition binding assays were performed using a range of heparin oligosaccharides to compete with polymeric heparin or heparan sulphate binding to I^125^ CXCL12. Heparin dodecasaccharides were found to be the minimal chain length required to efficiently bind CXCL12 (71% inhibition; *P*<0.001). These oligosaccharides also significantly inhibited CXCL12-induced migration of CXCR4-expressing LMD MDA-MB 231 breast cancer cells. In addition, heparin dodecasaccharides were found to have less anticoagulant activity than either a smaller quantity of polymeric heparin or a similar amount of the low molecular weight heparin pharmaceutical product, Tinzaparin. When given subcutaneously in a SCID mouse model of human breast cancer, heparin dodecasaccharides had no effect on the number of lung metastases, but did however inhibit (*P*<0.05) tumour growth (lesion area) compared to control groups. In contrast, polymeric heparin significantly inhibited both the number (*P*<0.001) and area of metastases, suggesting a differing mechanism for the action of polymeric and heparin-derived oligosaccharides in the inhibition of tumour growth and metastases.

Breast cancer is the most common malignancy in women and a major cause of morbidity and mortality in many parts of the world. The main cause of these deaths is metastatic spread of the disease (Dowsland *et al*, 2003). Breast cancer cells metastasise to specific anatomical sites, which include the brain, liver, lung and regional lymph nodes (Woodhouse *et al*, 1997). It appears that the metastatic spread of breast cancer cells to specific organs is dependant on the ability of these organs to express factor(s) that mediate specific cancer cell extravasation and recruitment from the blood, across the vascular endothelium and into the sub-endothelial tissue compartment. This process has clear parallels with the normal mechanism of leukocyte trafficking which is driven by chemokines (Muller *et al*, 2001).

Chemokines are small structurally related chemo-attractant cytokines that exert their effects locally in a paracrine or autocrine manner. Chemokines signal through G-protein-coupled receptors on cells inducing cytoskeletal rearrangement, firm adhesion to endothelial cells and directional migration (Zlotnik and Yoshie, 2000). These secreted proteins act in a co-ordinated fashion with cell-surface proteins, including integrins, to direct specific homing of various subsets of haematopoietic cells to specific anatomical sites (Moser *et al*, 2004).

There is increasing evidence that cancer cells express both chemokines and their receptors allowing both autocrine and paracrine responses, leading to migration, enhanced proliferation and cell survival (Azenshtein *et al*, 2002; Chen *et al*, 2006; Zlotnik, 2006). Indeed, metastatic breast cancer cells have been shown to express high levels of the chemokine receptor CXCR4 (Moore, 2001). In addition, organs commonly associated with being sites of breast cancer metastasis, such as lung, liver, lymph node, brain and bone, constitutively express the sole ligand for CXCR4, CXCL12 (Muller *et al*, 2001). Until recently, the interaction between CXCL12 and CXCR4 appeared to be unique and non-promiscuous. However, it has recently been argued that CXCL12 can interact with an additional receptor RDC1 (CXCR7), which is not thought to be involved in migration (Balabanian *et al*, 2005). Importantly, when signalling through CXCR4, CXCL12 stimulates intracellular calcium-flux and chemotaxis in lymphocytes, leukocytes and a range of cancer cells including breast (Amara *et al*, 1999; Fernandis *et al*, 2004). Although RDC1 is expressed by a number of cancer cell lines, it does not induce an intracellular calcium-flux or migration but may promote cell survival and adhesion (Burns *et al*, 2006).

The presentation of chemokines to their receptors *in vivo* is dependent on the glycosaminoglycan (GAG) components of cell surface or extracellular matrix proteoglycan molecules. Binding occurs between anionic, sulphated GAG residues and basic amino-acid domains within the chemokine primary structure (Ali *et al*, 2000). Mutagenesis studies have shown that GAG-binding is necessary before chemokines can stimulate normal cell migration *in vivo* (Ali *et al*, 2005; Proudfoot, 2006). It has been proposed that the immobilisation of chemokines by GAGs is required to support the stable, solid-phase chemokine concentration gradients necessary for leukocyte trafficking *in vivo* (Kuschert *et al*, 1999). Binding of chemokines to cell surface GAGs may also increase their local concentration (Hoogewerf *et al*, 1997), resulting in more sensitive signalling by specific chemokine receptors. Heparan sulphate is a polysulphated, highly negatively charged GAG synthesised in the Golgi, and is the predominant GAG present on the apical surface of vascular endothelial cells (Esko and Lindahl, 2001).

It is now thought that the metastasis of breast cancer cells is driven by the presentation of CXCL12 by heparan sulphate on the vascular endothelium and ECM. This induces the activation of integrins on CXCR4-expressing breast cancer cells which causes transendothelial migration of the cells followed by growth and angiogenesis resulting in secondary tumours (Beckmann *et al*, 1997; Murphy, 2001; Kucia *et al*, 2004; Luker and Luker, 2006). *In vivo* studies have confirmed the importance of CXCL12/CXCR4 interactions in breast cancer metastasis. Muller *et al* (2001) employed antibodies against CXCR4 in a model of breast cancer metastasis over 28 days, which dramatically reduced the incidence of lung metastases by 67%. A number of studies have investigated the knock down of CXCR4 using siRNA in breast cancer models. Liang *et al* (2005) demonstrated a significant decrease in the number of metastases in SCID mice inoculated with MDA-MB 231 cells transfected with CXCR4 siRNA compared to controls.

It is already known that the binding of chemokine to soluble heparan sulphate or heparin can inhibit the activation of leukocyte-borne chemokine receptors and prevent chemokine-dependant lymphocyte adhesion and migration (Kuschert *et al*, 1999; Ali *et al*, 2000; Sadir *et al*, 2001; Hecht *et al*, 2004). Heparinoids therefore provide a potential approach for inhibiting breast cancer metastasis, although their anticoagulant activity might complicate clinical application.

The current study was designed to determine the minimal chain length of a heparin oligosaccharide that could significantly inhibit CXCL12-mediated breast cancer metastasis, while abrogating the potential anticoagulation problems associated with the use of heparin as an antimetastatic treatment. A series of *in vitro* experiments were performed to determine the shortest length of oligosaccharide capable of competing CXCL12 from a solid-phase of heparin or heparan sulphate matrix and inhibiting breast cancer cell migration, while exerting little or no anticoagulant effect. Once the minimum length oligosaccharide was determined, *in vivo* experiments were designed to investigate the antagonistic effects of these oligosaccharides when compared to polymeric heparin on the haematological metastasis of CXCR4 expressing breast cancer cells. A final series of *in vitro* experiments were performed to define a candidate mechanism for the antimetastatic effect produced by heparinoids *in vivo*. We determined that a dodecasaccharide, an oligosaccharide composed of 12 monomeric sugar units (dp12) was the shortest length oligosaccharide able to significantly compete for CXCL12 binding against heparan sulphate *in vitro*. Dodecasaccharides exerted less anticoagulation activity than either polymeric heparin or the low molecular weight heparin Tinzaparin. Heparin significantly inhibited the formation of lung metastases *in vivo*. However, dp12 did not reduce the number of lung metastases, but did reduce the area of individual lesions.

## MATERIALS AND METHODS

### Preparation of oligosaccharides

Three 2 ml vials of the low molecular weight heparin product Tinzaparin (Leo Pharmaceuticals, Princes Risborough, UK), were pooled and applied to two Bio-Gel P10 columns (3 × 120 cm; Bio-Rad, Hamel Hempstead, UK) linked in series and eluted with 0. 25 M NH_4_HCO_3_ at a flow rate of 9 ml h^−1^. Tinzaparin is produced through the enzymatic degradation of polymeric heparin using the enzyme heparinase I (Linhardt and Gunay, 1999). Fractions of 3 ml were collected; their absorbance at 232 nm measured and relevant fractions pooled. After lyophilisation, the samples were dissolved at a concentration of 100 *μ*g *μ*l^−1^ before precipitation with three volumes of ice-cold ethanol containing 5% potassium acetate (Sigma, Poole, UK). The samples were centrifuged, the supernatant removed and the pellets left to dry overnight at room temperature before being weighed. This procedure typically yielded around 10 mg of oligosaccharide and was repeated as necessary to obtain sufficient quantities of oligosaccharide material. Fractions pooled to obtain dp24 and dp26 oligosaccharides are approximations as no clear peak corresponding to these size pools was directly detected within the size-exclusion profile of Tinzaparin.

### Radioligand binding assays

Scintillant-coated 96-well plates (Flashplate; PerkinElmer, Waltham, MA, USA) were treated overnight with 200 *μ*l of 0.25 mg ml^−1^. Poly-L-lysine hydrobromide (PLL; Sigma) in PBS. Following washing with 0.01 M PBS, the wells were incubated with 250 *μ*l of heparin (H3400; Sigma) or heparan sulphate (Sigma) at a concentration of 50 *μ*g ml^−1^ in PBS for 2 h at 37°C; negative control wells contained only PBS. The wells were then washed twice in binding buffer (1 mM CaCl_2_, 5 mM MgCl_2_, 0.5% BSA, 50 mM HEPES; pH 7.2). Soluble competitor was added in a volume of 150 *μ*l per well at desired concentrations followed by the addition of 5 nM unlabelled CXCL12 (PeproTech EC LTD, London, UK) with a tracer concentration of 100 pM ^125^I-CXCL12 (PerkinElmer) in a volume of 50 *μ*l in binding buffer. The plates were incubated at room temperature for 3 h with shaking and the solution was removed from each well. After washing three times with 200 *μ*l of binding buffer the bound radioactivity was measured in a scintillation counter (Microbeta; LKB Wallac, PerkinElmer, Waltham, MA, USA).

### Chemotaxis assays

LMD MDA-MB 231 cells were assayed for their ability to migrate through an 8 *μ*m pore diameter filter membrane (Falcon culture inserts; Becton Dickinson, Cowley, UK) towards 12.5 nM CXCL12. 200 000 cells were placed in the upper chamber and the lower chamber was loaded with medium containing 12.5 nM CXCL12 and varying concentrations of dp12. The assay was incubated for 8 h at 37°C before removal of excess cells and medium from both chambers. The upper surface of each filter was gently swabbed before removal and fixation in methanol at 0°C for 1 h. The filters were then stained with 0.1% Mayer's haematoxylin (Sigma) for 10 min, followed by a 10 min wash in Scott's tap water (166 mM MgSO_4_, 24 mM NaHCO_3_). Finally, the filters were sequentially dehydrated with 50, 75, 90 and 100% ethanol, air-dried and mounted. Migrant cells on each filter were identified by high-power microscopy (× 40) and counted in nine randomly selected fields; the operator was blinded to the treatments.

### Anti-coagulation assay

The anti-coagulation effects (anti-Xa activity) of heparin (2.2 *μ*g ml^−1^), Tinzaparin (3.4 *μ*g ml^−1^; Leo Pharmaceuticals), and dp12 oligosaccharides (0–34 *μ*g ml^−1^) were examined in plasma. Whole blood mixed with an appropriate heparinoid was added to citrated blood collection tubes and immediately analysed. Briefly, the plasma was centrifuged at 3000 *g* for 20 min and transferred to fresh tubes. The anti-Xa level of each sample was then assessed using the Biophen heparin 6 kit (Hyphen BioMed, Neuville-sur-Oise, France) and the COAG-A-MATE MTX II (BioMerieux, Marcy l’Etoile, France) coagulation analyser. Each batch of samples was analysed in conjunction with Biophen low molecular weight heparin (LMWH) control plasma and Biophen heparin control plasma (Hyphen Biomed) as standards.

### *In vivo* metastasis study

All animal experiments were performed in accordance with local ethical review committee and Home Office Project Licence (PPL 60/3375) approval. All reasonable efforts were made to minimise the suffering likely to be caused and the number of animals to be used. All animals were inspected daily by a qualified technician and weighed twice weekly to ensure thorough assessment of the health of all animals involved.

CB-17 severe combined immunodeficient (SCID) mice (Charles River Labs, Wilmington, MA, USA) were injected subcutaneously with 3.3 mg kg^−1^ day^−1^ (600 IU kg^−1^ day^−1^) of heparin in PBS every 12 h, once a day with dp12 in PBS (4.0 mg kg^−1^ day^−1^) or with PBS alone. Seven mice were treated in each group. Four hours after the first PBS/heparinoid injection, mice were injected with 2 × 10^5^ LMD MDA-MB 231 breast carcinoma cells into the tail vein. Lungs were collected on day 28 and fixed for histopathology. Twenty sections were cut from each mouse lung at intervals of 50 *μ*m in depth.

Lung sections were assessed blindly at × 10 magnification (Leica DMR) and the area of metastasis was calculated using Leica Q-Win software. Both the number and area of the metastatic lesions were assessed blindly by two separate investigators (Paul Mellor and James Harvey).

### CXCL12 immunohistochemistry of frozen tissue

Serial sections (5 *μ*m thick) of mouse lung were fixed in acetone for 10 min at room temperature and then air dried. Following a wash for 5 min in tris-buffered saline (TBS), endogenous peroxidase activity was blocked by incubating for a further 5 min in 0.6% H_2_O_2_ in methanol. Sections were then rinsed in distilled water and washed for a further 5 min in TBS; antigen retrieval was not necessary. Endogenous avidin and biotin were blocked (SP-2001; Vector Labs, Peterborough, UK). Non-specific binding of the secondary antibody was prevented by incubation of the sections with 20% rabbit's serum in TBS for 1 h at 4 °C. Sections were then incubated overnight at 4°C with primary antibody anti-CXCL12 (Mab310; Sigma) at a concentration of 1/20 in 20% rabbit's serum/TBS solution. Two control sections per tumour were also incubated in every run of immunohistochemistry; these sections were incubated overnight with no primary antibody (20% rabbit's serum/TBS alone). Following washing in TBS, sections were incubated for 1 h at room temperature with a rabbit-anti-mouse biotinylated secondary antibody (1/250; Dako, Ely, UK). After a further wash step in TBS, sections were incubated for 30 min in the dark with a Streptavidin ABC complex (Vectastain ABC; Vector Labs) at room temperature. A final wash step was performed before incubation with NiDAB (SK-4100 substrate kit; Vector Labs) for visualisation.

Light counterstaining with Haematoxylin (Sigma) was performed on all specimens before mounting; this enabled visualisation of the tissue architecture without masking of the NiDAB staining. Sections unstained with antibody were dual stained with Haematoxylin and Eosin (H&E) to demonstrate the tissue architecture in serial sections. Slides were dehydrated through a series of alcohol baths (50, 70, 90, 95 and 100% alcohol), cleared in xylene and mounted using dibutylphthalate xylene. Sections were compared with controls, where lungs were stained with an isotype control instead of an anti-CXCL12 primary antibody.

### CXCR4 real-time RT-PCR

Total RNA was isolated from murine lung tissue using Trizol (Life Technologies, Paisley, UK) and cDNA was generated using oligo-dT primers with the Superscript II RNase reverse transcription system (Invitrogen, Paisley, UK). Real-time TaqMan PCR was performed according to Applied Biosystems protocols. All samples were loaded in triplicate wells and fluorescence emission detected by the ABI Prism 7000 (Applied Biosystems, Foster City, CA, USA). Primers and probes for CXCR4 were purchased from Applied Biosystems. All results were normalised to the expression of endogenous glyceraldehydes-3-phosphate dehydrogenase mRNA and expressed as a relative increase or decrease in expression compared to PBS controls.

### Statistical analysis

Data values for radioligand binding, chemotaxis assays and *in vivo* metastasis model were expressed as means±s.e.m. Statistical analysis was performed using an unpaired two-tailed Student's *t*-test or using a one-way analysis of variance when multiple data sets are being compared; *P*<0.05 was considered significant.

## RESULTS

### Determination of shortest-length oligosaccharide of heparin capable of significantly competing CXCL12 from heparan sulphate

**Figure 1:**
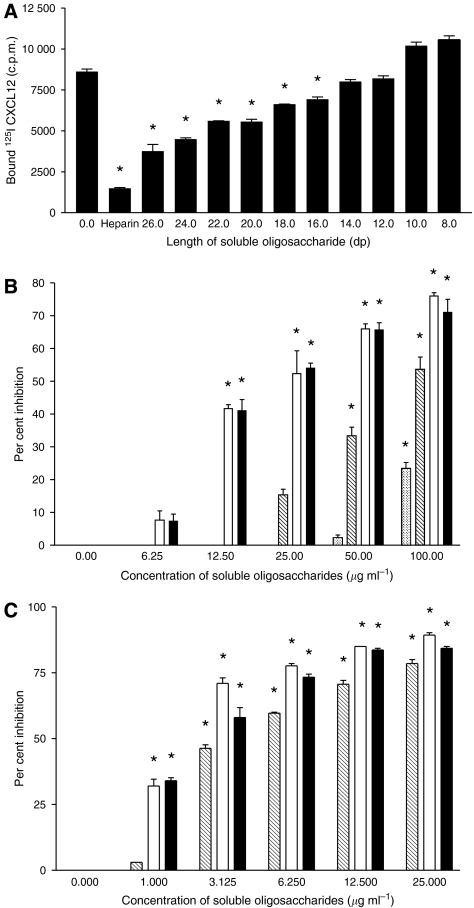
The shortest length oligosaccharide of heparin capable of significantly competing ^125^I-CXCL12 from solid-phase heparin or heparan sulphate (50 *μ*g ml^−1^) was determined using radioligand competition binding assays. (**A**) Initially a range of soluble oligosaccharides between 26 and 8 monosaccharide units in length and heparin (7 *μ*g ml^−1^), were assessed with solid-phase heparin (50 *μ*g ml^−1^). The oligosaccharides dp14 (filled), dp12 (open), dp10 (hashed) and dp8 (dots) were then (**B**) used at a range of concentrations to compete CXCL12 from heparin. Finally (**C**), the dp14, dp12 and dp10 oligosaccharides were competed against a heparan sulphate solid-phase for CXCL12 binding at a range of concentrations (^*^P<0.001). Data are representative of three individual experiments; bars show mean values±s.e.m.

Radioligand binding assays were performed to determine the potential of varying concentrations of soluble heparin and heparin oligosaccharides to compete with polymeric heparin or heparan sulphate to bind ^125^I-CXCL12. Initially, polymeric heparin and the oligosaccharides dp26–dp8 were used at a constant concentration of 7 *μ*g ml^−1^ (representative molar concentrations: dp26–0.9 *μ*M, dp8–3*μ*M), determined from the IC_50_ of the dp26 oligosaccharides (results not shown), to compete CXCL12 from a solid phase of heparin. Polymeric heparin and the oligosaccharides dp26–dp16 all competed significantly (*P*<0.001; Figure 1A) with solid-phase heparin, whereas the competition exerted by dp14 and 12 (7 and 5% respectively) was not significant (*P*>0.05). As the length of the oligosaccharides decreased so did the level of competition seen. Unexpectedly, the oligosaccharides dp10 and 8 caused a small increase in binding of CXCL12 to solid-phase heparin.

When analysed over a range of concentrations both the dp14 and 12 oligosaccharides significantly competed with solid-phase heparin at similar low concentrations (12.5 *μ*g ml^−1^; *P*<0.001), whereas the dp10 and 8 oligosaccharides were required at much higher concentrations (50 *μ*g ml^−1^ and in excess of 100 *μ*g ml^−1^ respectively) to achieve similar levels of competition (40% inhibition; Figure 1B). The ability of the dp14, 12 and 10 oligosaccharides to compete CXCL12 from a more biologically relevant solid phase of heparan sulphate was also measured. As with the previous experiment both the dp14 and 12 oligosaccharides significantly competed for CXCL12 at similar low concentrations (1 *μ*g ml^−1^), but higher concentration of the dp10 oligosaccharides were required to exert the same level of competition (Figure 1C).

### Assessment of the effects of dp12 on breast cancer cell migration *in vitro*

**Figure 2:**
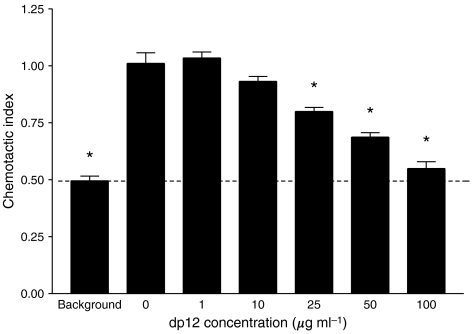
The effects of dp12 on the migration of breast cancer cells. The number of LMD MDA-MB 231 cells migrating through 8 *μ*M pores towards 12.5 nM CXCL12 was measured in the presence of varying concentrations of the dp12 inhibitor (^*^P<0.001). The results are expressed as a chemotactic index normalised to the number of migrating cells in the presence of CXCL12 with no dp12. Data are representative of three individual experiments; bars show mean values±s.e.m.

Following definition of dp12 as the shortest oligosaccharide to significantly bind CXCL12, this size pool was assayed for its effect on breast cancer cells *in vitro*. It was found that the migration of breast cancer cells across transmembrane filters towards 12.5 nM CXCL12 was significantly inhibited in the presence of 25 *μ*g ml^−1^ (7 *μ*M) of the dp12 oligosaccharide (*P*<0.001; Figure 2). In the presence of 100 *μ*g ml^−1^ of dp12 the number of migrant cells was similar to the background level with no chemokine (*P*>0.05).

### Analysis of the anticoagulant potential of heparinoids

**Figure 3:**
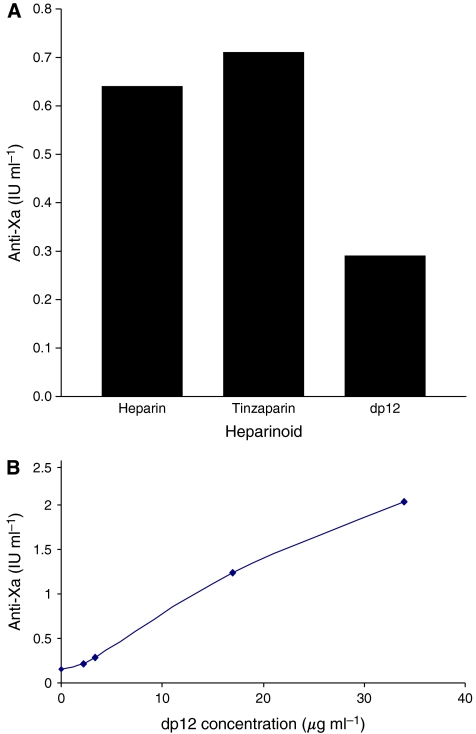
The anticoagulation properties of dp12. (**A**) Anti-Xa levels in plasma isolated from whole blood receiving doses of heparin, tinzaparin and an equivalent dose (weight) of dp12; therapeutic levels are between 0.5–1.0 anti-Xa IU ml^−1^. (**B**) Anti-Xa levels in whole blood containing varying doses of dp12.

An important aim of this study was to identify heparin oligosaccharides that possess less anticoagulant activity than the polymeric heparin molecule from which they are derived. To achieve this anti-Xa levels were assessed in plasma isolated from whole blood that had received doses of heparin (2.2 *μ*g ml^−1^), Tinzaparin, a LMWH with a similar average molecular weight to dp12 (3.4 *μ*g ml^−1^) and dp12 (3.4 *μ*g ml^−1^; 1 *μ*M) (Figure 3A). The doses of both heparin and Tinzaparin were given to achieve plasma concentrations (0.2–0.4 IU ml^−1^) that correspond to what is clinically known as a therapeutic dose, with therapeutic anti-Xa levels known to be in the range 0.5–1.0 anti-Xa IU ml^−1^. As there was no known therapeutic dose of dp12 the same concentration (weight) as that used for Tinzaparin was used. Both heparin and Tinzaparin-treated blood showed anti-Xa IU ml^−1^ values within the therapeutic range (0.64 and 0.71 anti-Xa IU ml^−1^) respectively. However, the anti-Xa value for dp12-treated blood was 0.29 IU ml^−1^, which is below therapeutic values. As the concentration of dp12 increased so did the anti-Xa IU ml^−1^ values, indicating that dp12 is an effective anticoagulant at high concentrations (Figure 3B).

### Determination of the effects of heparin and dp12 on breast cancer metastasis *in vivo*

LMD MDA-MB 231 cells (2 × 10^5^) were administered intravenously to three groups of seven SCID mice on day 1. The mice were independently treated from days 0 to 28 with either subcutaneous doses of heparin (3.3 mg or 0.17 *μ*mol kg^−1^ day^−1^; 600 IU kg^−1^ day^−1^) given twice daily, 0.1 ml PBS given once daily, or dp12 (4.0 mg or 1.2 *μ*mol kg^−1^ day^−1^) also given once daily. This dose of heparin is known to produce therapeutic anticoagulation in a clinical setting. Treatment of SCID mice with a single daily dose of Tinzaparin at 175 IU kg^−1^ day^−1^ (1.8 mg or 0.6 *μ*mol kg^−1^ day^−1^) produces a level of anticoagulation, which is just within the therapeutic range (0.51 anti-Xa IU ml^−1^); data not shown. Given that Tinzaparin has an anticoagulant activity, which is approximately 2.5 times greater than that of dp12, a sub-anticoagulant dose of dp12 was chosen to dissociate anticoagulant activity from any putative anticancer effect. The half-life of LMWHs is two to four times that of unfractionated heparin; for this reason, typical clinical regimes involve administration of LMWHs once a day and heparin twice a day (Hirsh and Levine, 1992; Wu, 2001). As both dp12 and LMWHs are the result of polymeric heparin degradation, dp12 was also administered once a day.

**Figure 4:**
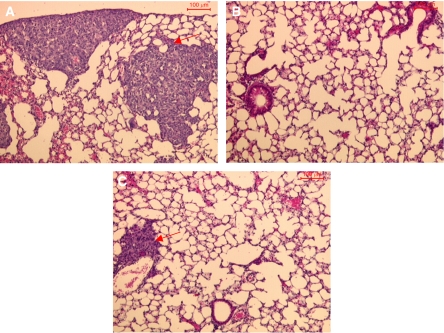
Representative haemotoxylin and eosin staining of tumours (red arrows) in animals treated with (**A**) PBS, (**B**) heparin and (**C**) dp12 for 28 days after intravenous administration of LMD MDA-MB 231 cells.

**Figure 5:**
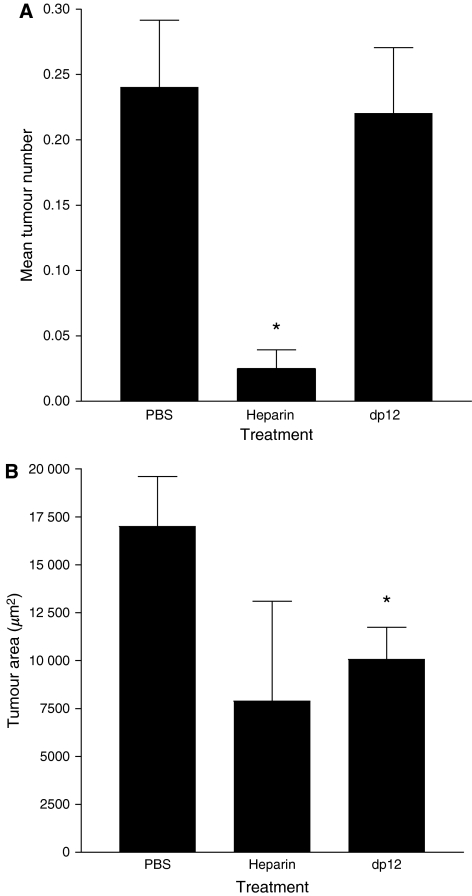
Murine breast cancer model. Following administration of LMD MDA-MB 231 cells, three groups of seven mice were treated for 28 days with subcutaneous doses either of heparin (3.3 mg kg^−1^ per day) given twice daily, 0.1 ml PBS given once daily, or dp12 (4.0 mg kg^−1^ per day) also given once daily. A total of 20 sections from each mouse lung were H&E stained and assessed blindly using Leica Q win software for both the number (**A**) of metastases and the area (**B**) of each metastasis identified (^*^P<0.001); bars show mean values±s.e.m.

A total of 20 sections from each mouse lung were assessed for both the presence and the area of each metastatic lesion (Figure 4). Dp12 had no effect upon the number of breast cancer metastases found in the lungs when compared to control PBS-treated mice (Figure 5A). However, the metastases within the lungs of dp12 treated mice were on average 41% smaller (*P*<0.05) than those found in the PBS control mice (Figure 5B). Heparin decreased the number of metastases by 87% (*P*<0.0001) compared to the PBS-treated group (Figure 5A). No significant difference was identified between the area of metastases in the heparin and the PBS-treated groups (Figure 5B). Only three metastases were identified in the heparin-treated animals (*n*=7), therefore giving a large s.e.m. for the average size of the lesions identified.

### Assessment of the expression of CXCL12 on the lung vascular endothelium and of CXCR4 on the formed metastases

**Figure 6:**
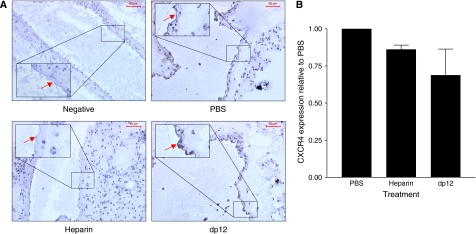
CXCL12 and CXCR4 expression in mouse lung and tumours. CXCL12 expression on the surface of the vascular endothelium was assessed by immunohistochemical staining using a CXCL12 mouse monoclonal antibody (**A**) in mouse lung treated with PBS, heparin and dp12 for 28 days following administration of LMD MDA-MB 231 cells (red arrows); negative section is isotype control. There was strong CXCL12 staining on the vascular endothelium in the PBS-treated group, which was unaltered in the dp12 group, whereas CXCL12 was absent from the vascular endothelium of the heparin-treated mice. The levels of human CXCR4 expression (**B**), relative to PBS group, in tumours present in the mouse lung treated with heparin and dp12 for 28 days following administration of LMD MDA-MB 231 cells were assessed using real-time PCR normalised to human GAPDH. Data are representative of three individual experiments; bars show mean values±s.e.m.

Both the expression of CXCR4 on cancer cells and CXCL12 on the apical surface of vascular endothelium were examined to assess the role played by chemokines in the anticancer activity of heparinoids. The surface of normal lung endothelium expressed a high level of CXCL12, which was not altered in PBS-treated control animals (Figure 6A). Treatment with dp12 slightly decreased CXCL12 expression while none of this chemokine was detected on lung endothelium from heparin-treated mice (Figure 6A). Tumours in both the heparin and dp12-treated mice showed a small, but non-significant (*P*>0.05), decrease in the expression of CXCR4 mRNA in comparison with PBS-treated animals (Figure 6B).

## DISCUSSION

The metastasis of breast cancer cells to specific sites in the body is the main cause of death for patients with this disease (Walker *et al*, 1997). Recent evidence has shown that this metastasis is mediated through specific cell migration stimulated by the interactions of CXCL12, presented by heparan sulphate on the vascular endothelium of organs commonly associated with breast cancer metastasis, with CXCR4, expressed at high levels on metastatic breast cancer cells (Muller *et al*, 2001). Previous studies have shown that heparin is capable of antagonising chemokine function both *in vitro* and *in vivo* (Castelli *et al*, 2004) indicating its potential to disrupt the interaction between CXCL12 and CXCR4, thereby preventing the invasion of CXCR4 expressing breast cancer cells (Dowsland *et al*, 2003). Clearly, the anticoagulant properties of heparin might limit the usefulness of this molecule in treating breast cancer patients, especially peri-operatively. This study has identified a short-chain length oligosaccharide derived from polymeric heparin capable of inhibiting the migration of breast cancer cells *in vitro* and limiting the growth of secondary tumours *in vivo*, while minimising potential anticoagulation problems associated with heparin.

The potential of defined-length heparin oligosaccharides to compete with solid-phase heparin to bind CXCL12 was found to decrease as the chain length decreased. Soluble heparin and the oligosaccharides ranging from dp26 down to 16 in length all competed significantly with solid-phase heparin, while oligosaccharides below 16 monomeric sugar units in length failed to compete. In all cases, a similar amount (7 *μ*g ml^−1^) of defined-length GAG was used to compete with heparin for chemokine binding, suggesting that oligosaccharides containing 14 or fewer monosaccharide units have a reduced potential to bind CXCL12. However, heparinoids below dp14 did show some potential to bind CXCL12 when present at greatly increased concentrations. The most likely explanation for this observation is a reduction in the number of the chemokine binding domains as the chain length of the heparinoid molecule is reduced.

CXCL12 exists as a monomer in solution but forms dimers when interacting with heparin or heparan sulphate (Veldkamp *et al*, 2005). The stoichiometry of the binding of CXCL12 to GAGs suggests that each chemokine occupies an average of six monosaccharide units on heparin (Altundag *et al*, 2005). However, Sadir *et al* (2001) have shown that 13 monosaccharide units are required for interaction with the dimeric form of CXCL12. This is consistent with data in the current study and potentially explains the greater degree of competition seen with dp14 and 12 than with dp10 and 8 as the longer species are capable of binding dimeric CXCL12, whereas the shorter can only bind monomeric CXCL12. For these reasons, dp12 was chosen for further study.

Cell surface GAGs play important roles in the presentation of chemokines to their specific receptors; these include protection of chemokine molecules from degradation and the maintenance of the stable concentration gradients through the tissues which are required for vectorial cell migration (Perrimon and Bernfield, 2000). However, soluble GAGs have also been demonstrated to antagonise the biological activity of chemokines (Douglas *et al*, 1997) potentially by production of chemokine-GAG complexes which cannot engage specific chemokine receptors (Ali *et al*, 2000). The current study confirmed these observations by demonstration that dp12 inhibits the migration of breast cancer cells towards CXCL12 *in vitro*.

The dp12 oligosaccharide had significantly less anticoagulant activity than either heparin or Tinzaparin. As a consequence, higher doses of dp12 were required to produce a similar level of anti-Xa activity in blood. This can be explained in terms of the relative length of the molecules. It is known that specific pentasaccharide sequences are required for AT binding; these motifs are present in about a third of heparin molecules (Lindahl *et al*, 1979). However, only between 15 and 25% of the shorter fragments present in LMWH contain this sequence (Lindahl *et al*, 1979; Lane *et al*, 1984; Hirsh, 1998; Herault *et al*, 2002). The variation in the occurrence of the pentasaccharide between LMWHs results in differences in their ability to bind AT and therefore in inhibiting factor Xa (Nader *et al*, 2001). The cleavage of heparin required to produce oligosaccharides is likely to disrupt many of these sequence motifs, reducing the number available for AT binding within a given weight of the processed material. Importantly, however, the oligosaccharides retained their potential to antagonise chemokine activity; this is consistent with previous data from our group of the separation of anticoagulant and chemokine-binding functions.

An *in vivo* breast cancer model was used to determine the ability of heparin to prevent the metastasis of human breast cancer cells from peripheral blood to the lung tissue of SCID mice; importantly, heparin powerfully reduced the number of metastatic lesions observed in the lung while treatment with the dp12 oligosaccharides had no effect on the number of lesions. Further experiments were performed to investigate the possible role of chemokine antagonism in this model. Specifically, it was found that treatment with heparin significantly reduced the expression of CXCL12 on the endothelial and epithelial surfaces in the lung, while dp12 only produced a small decrease in CXCL12 expression. This suggests that heparin therapy reduces the potential of CXCL12 to ‘capture’ cancer cells within breast tissues. However, no reduction in the ratio between mRNA sequences encoding human CXCR4 and GAPDH was observed in any treatment group suggesting that the absence of immobilised CXCL12 in heparin-treated animals did not select for non-CXCR4-expressing variants of the cancer.

The tumours in the dp12-treated group were significantly smaller in size than those in the PBS-treated control group. Tumour growth is dependent not only on growth factors produced by the cancer cells themselves but also those present in the surrounding microenvironment. Heparinoids are very anionic molecules that can bind and modulate the function of a wide range of cytokines and adhesion molecules (Dowsland *et al*, 2003), inducing numerous changes in the tissue microenvironment with the potential to affect cancer cell growth. It is therefore most probable that dp12 is affecting factors other than CXCL12 in mediating its effects on tumour growth. For example, heparin derivatives as short as eight monosaccharide units can bind both FGF-2 and VEGF *in vivo* (Ishihara *et al*, 1993; Tyrrell *et al*, 1993; Hurwitz *et al*, 2004); this prevents these growth factors from binding to heparan sulphate leading to inhibition of angiogenesis (Hasan *et al*, 2005). As these oligosaccharides prevent FGF-2 and VEGF from binding to heparan sulphate, they function as antagonists of angiogenesis and have been shown *in vivo* to inhibit the development of new vasculature (Hasan *et al*, 2005).

However, it is important to stress that the action of heparin in inhibiting breast cancer metastasis is potentially multifactorial, and not solely the result of the inhibition of CXCL12/heparan sulphate interactions. Studies examining the role of soluble heparin in breast cancer have demonstrated a number of mechanisms vital for breast cancer metastasis that can be disrupted by heparin. These include the inhibition of the adhesion and migration of breast cancer cells by heparin-mediated inhibition of selectin binding and proteases These processes are influenced to a greater degree by heparin than LMWHs and may potentially explain the differences seen between heparin and dp12 (Vlodavsky *et al*, 1994; Koenig *et al*, 1998; Borsig, 2004). In addition, if dp12 does function by inhibiting the action of growth factors on the breast cancer cells it is unlikely that heparin will be functioning in a similar manner with studies demonstrating a promotional role for heparin in the presentation of growth factor receptors, in contrast to shorter length oligosaccharides (Soker *et al*, 1994; Norrby and Ostergaard, 1996).

In conclusion, these data show that relatively non-anticoagulant concentrations of a soluble dp12 oligosaccharide can significantly inhibit the migration of breast cancer cells *in vitro*, but only inhibit the growth and not the number of lung metastases which form *in vivo.* However, anticoagulant concentrations of heparin can significantly inhibit both *in vitro* migration and the metastatic spread of breast cancer cells *in vivo*. As viable cancer cells are liberated into the vasculature during surgical resection, heparin may offer a potential antimetastatic therapy. Although clinically dp12 would not be applicable as an antimetastatic therapy it may prove useful in inhibiting the growth of established tumours, offering a potential treatment following diagnosis and before surgery. The oligosaccharide may also prove beneficial to patients with non-operable tumours by extending survival times.
